# Emergence of a coherent and cohesive swarm based on mutual anticipation

**DOI:** 10.1038/srep46447

**Published:** 2017-04-13

**Authors:** Hisashi Murakami, Takayuki Niizato, Yukio-Pegio Gunji

**Affiliations:** 1 Kanagawa University, Department of Information Systems Creation, Yokohama, 221-8686, Japan; 2 Tsukuba University, Faculty of Engineering, Information and Systems, Tsukuba, 305-8571, Japan; 3 Waseda University, School of Fundamental Science and Engineering, Shinjuku, 169-0072, Japan

## Abstract

Collective behavior emerging out of self-organization is one of the most striking properties of an animal group. Typically, it is hypothesized that each individual in an animal group tends to align its direction of motion with those of its neighbors. Most previous models for collective behavior assume an explicit alignment rule, by which an agent matches its velocity with that of neighbors in a certain neighborhood, to reproduce a collective order pattern by simple interactions. Recent empirical studies, however, suggest that there is no evidence for explicit matching of velocity, and that collective polarization arises from interactions other than those that follow the explicit alignment rule. We here propose a new lattice-based computational model that does not incorporate the explicit alignment rule but is based instead on mutual anticipation and asynchronous updating. Moreover, we show that this model can realize densely collective motion with high polarity. Furthermore, we focus on the behavior of a pair of individuals, and find that the turning response is drastically changed depending on the distance between two individuals rather than the relative heading, and is consistent with the empirical observations. Therefore, the present results suggest that our approach provides an alternative model for collective behavior.

Mobile animal groups such as fish schools and bird flocks exhibit spontaneous polarized movement patterns. Many theoretical models have been proposed on how a collective order pattern would result from local interactions^1^,^2^,^3^,^4^,^5^,^6^,^7^,^8^. The basic assumption incorporated into these models was the alignment rule, according to which an agent matches its velocity with those of others in its neighborhood. The self-propelled particles model (SPP)^9^, in particular, which was inspired by the emergent collective properties of the physical system, is commonly used to explain collective behavior. The SPP model exhibits a phase transition by combining the alignment rule with external noise.

With the advent of tracking and bio-logging, through the advance of image analysis techniques and global positioning systems, both empirical and observational kinetic data from members of real animal groups have been accumulated. These data enable the investigation of both the individual^10^,^11^,^12^,^13^,^14^,^15^ and group-level^16^,^17^,^18^,^19^,^20^,^21^,^22^,^23^ behaviors within real groups. Ballerini *et al*.^11^ showed that individuals in a starling flock use topological distance rather than metric distance as considered in most previous models. In addition, through simulation, they demonstrated that a flock based on topological distance is more robust to a predator’s attack than a flock based on metric distance (refer to ref. 21 for the preference of metric distance in other organism under different circumstances). Cavagna *et al*.^17^ revealed that the internal structures of collective animal groups are not stable in time. Rather, individuals move super-diffusively within their group and change their relative position with their neighbors, even though they appear to exhibit cohesive synchronized behavior. Furthermore, Murakami *et al*.^15^ showed that such an internal movement within a fish school is not merely erroneous random motion derived from alignment interactions among individuals but rather dynamically self-organized in both time and space.

Empirical data obtained from real animals have been analyzed applying theoretical models. Buhl *et al*.^16^ showed that the phase transitions in aggregating locusts from disordered to ordered patterns depends on their density, and that this behavior can be mimicked by the SPP. It was also reported that even in a generic mean-field approximation of the dynamics, where the interaction does not need to be fully specified, the same results are obtained^24^. Berdahl *et al*.^20^ revealed that, as a group increases in number, it responds more efficiently to environmental stimuli. Namely, a fish school prefers a darker area similar to their habitat and avoids the light. Larger schools are more sensitive to light. Berdahl *et al*.^20^ explained this emergent behavior by a simple rule based on the attraction between an agent and its neighbors. Thus, previous models showed good agreement with the experimental and observational data.

However, a conflict was reported between empirical data and previous models, particularly regarding the alignment rule. Katz *et al*.^13^ analyzed the free-swimming behavior in fish schools consisting of two or three individuals. They inferred how individuals interact with a single neighbor, without assuming that they coordinate their responses with multiple neighbors. Based on the fish’s acceleration, they decomposed its force into speeding and turning forces that were tangential and perpendicular to the velocity, respectively. If members of a collective group actually implemented the alignment rule, the turning force would increase when the relative angle between their velocities became larger. On the contrary, they observed that the strength of the turning force changes depending on the neighbor’s position rather than on the relative angle. They suggested that there is no direct alignment rule, and that the polarized pattern resulted from interactions, such as attraction and/or repulsion, that do not follow the explicit alignment rule.

Several researches have reported collective motions resulting from attraction-repulsion interaction without the alignment^25^,^26^,^27^,^28^,^29^,^30^. For example, active Brownian agents that interact globally with each other by attraction show noise-induced transition between translational and rotational motions^25^,^26^. Moreover, collective rotational or translational motion also results from springlike attraction-repulsion interaction with equilibrium distance without alignment^27^. Furthermore, the pursuit-escape interaction motivated by non-cooperative behavior such as cannibalism as a driving force of collective motion in locusts would be an alternative to the direct alignment^28^,^29^. In this model, agents pursuit their neighbors who are moving away from them and/or escape from those who are approaching them, depending on the relative velocity. This model shows the emergence of global polarization. But at least if both pursuit and escape interactions are implemented it would be unsuitable for the experimental results with respect to the social turning force in ref. 13 because in this model the direction of turning depends on the relative velocity between individuals (i.e., whether they are approaching or moving away), that is, it would be not always toward the neighbor’s position (but see also pursuit-only and escape-only models and their different collective patterns^28^,^29^). To our knowledge, so far no theoretical models did show direct correspondence to the results with respect to the social turning force in ref. 13.

In the present study, we show a local interaction named by mutual anticipation that is inspired the behavior of a swarm of soldier crabs^31^,^32^,^33^,^34^,^35^. We previously proposed a model based both on the alignment and the mutual anticipation with asynchronous position updating^36^,^37^. In the model, each individual is assigned a distinct vector (called the principal vector) and multiple vectors (representing potential transitions) that are randomly distributed from the principal vector within a maximal angle and maximal length. Individuals asynchronously move to popular sites with respect to the potential transitions among individuals, follow the antecessors (neighbors moved to next sites ahead of them), or move freely by choosing one of the potential transitions. Then, the alignment rule is applied to the principal vector within the neighborhood. We found that this model of a swarm is more robust against external perturbations than previously considered models^34^. Moreover, we revealed that an emergent behavior of the soldier crabs could be explained by mutual anticipation^31^. In addition, we showed that a robust mobile swarm without collective order could be simulated using only on mutual anticipation without velocity matching and the randomly modified principal vector^35^. Recently several researches have also reported collective motions resulting from anticipation. For example, when anticipation is implemented by angular velocities of neighbors (i.e., anticipation of neighbors’ orientation changes), and is coupled with the alignment rule and angular noise, it can yield various collective motions such as flocking, spinning, and swarming^38^. Moreover, when anticipation is implemented by linear predictions of neighbors’ future positions by their current velocities, and is incorporated in attractive-repulsive interaction, it leads rapid formation of a milling pattern in a wide range of prediction time^39^.

The present study describes a model based only on mutual anticipation, and not on the alignment rule. The agent in our model matches its principal vector with its own previous velocity, while the agent in most previous models that incorporates the explicit alignment rule matches its velocity with the neighbors’ velocities. We show that this model forms a dense swarm with high polarity, despite the absence of the explicit alignment rule. Furthermore, we investigate the behavior of individuals in two-individual swarms and observe that their turning force is drastically changed owing to the distance between them rather than the relative angle. This result is consistent with previous empirical data^13^.

## Results

### Swarm model

**Figure 1:**
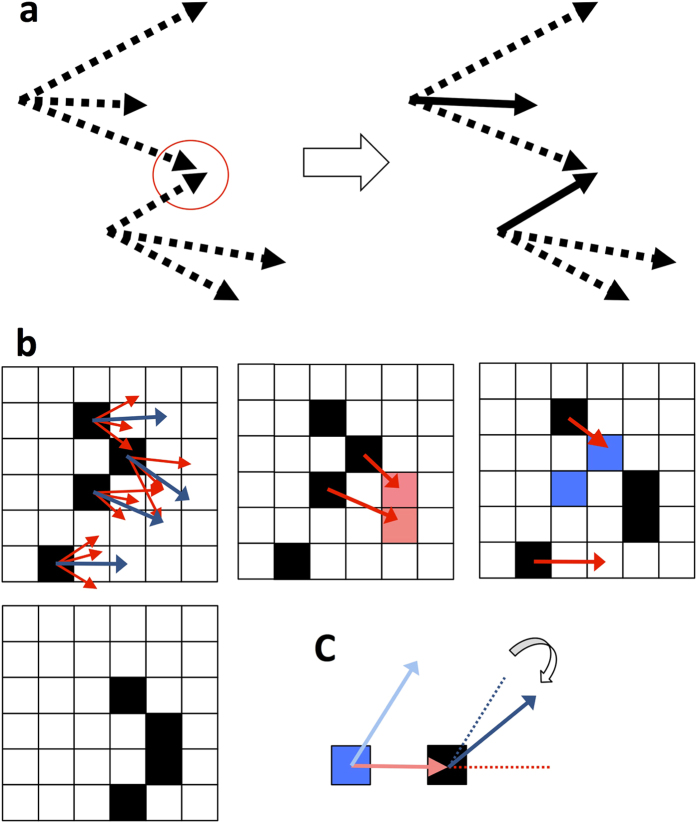
Schematic diagrams of transition of our model. (**a**) The essential structure of mutual anticipation. Each individual in our model has multiple potential transitions. In this figure there are two individuals who each have three potential transitions (dashed arrows). By the potential transitions individuals anticipate the movements each other and thereby intend to move to a site represented by a red circle in this figure where targets of the transitions are overlapped (left). Then one of individuals moves to the site, and the other avoids there and asynchronously moves by the remaining potential transitions (right). The resulting moves are represented by solid arrows. (**b**) Transition of asynchronous update. Distribution of potential transitions (upper left), Mutual anticipation (upper second left), following and free move (upper right), and the resulting distribution (bottom). Individuals are represented by black lattices. Principal vectors with *L* lattice length are represented by a blue arrow and *P* potential transitions are represented by a red arrow (*P* = 4 and *L* = 2 in the figure). The popular sites and the created empty sites are represented by pale red and pale blue lattices, respectively. (**c**) Update of the principal vector. After an individual updates its position from a pale blue square to a black square (its velocity represented with a pale red arrow), its principal vector is updated from a pale blue arrow to a blue arrow. At this time, *ω* determines the directional persistence of the principal vector (see also main text and Supplemental Information, Appendix S1 and Figure S1).

In this section, we introduce our model based on mutual anticipation and asynchronous updating, inspired by the behavior of soldier crabs. In our model, position update of each individual at a time step is asynchronously implemented by either of three rules (mutual anticipation, following or free moving). The mutual anticipation is the most important mechanism for our model and the other rules (following and free moving) perform backup roles (for the proportion of each rule implemented by individuals in our model, see Supplemental Information, Figure S2. See also ref. 31 for other roles of following in our model). The essence of the mechanism is that (i) each individual has multiple potential transitions by which it anticipates the movements of other individuals; (ii) if there are sites where targets of potential transitions are overlapped, one of individuals whose potential transitions reach the site moves there; (iii) then the other individuals avoid the site and asynchronously move by the remaining potential transitions (Fig. 1a). After position updates by mutual anticipation and the other rules (i.e., following and free moving), velocities of individuals are updated. In the rest of the section, we first describe how these rules are inspired by behavior of a swarm of soldier crabs. Next we describe in due order how asynchronous position update is implemented, explaining about each rule. Finally we describe how individuals’ velocities are update.

#### Collective behavior of a swarm of soldier crabs

Here we describe how our model is inspired by behavior of a swarm of soldier crabs. Through numerical field observations and experimental results^31^, we identified the following characteristics of general swarming behavior in soldier crabs: (i) a swarm moving in the tidal zone has inherent noise, i.e., individuals have different velocities in maintaining a directed group; and (ii) when a swarm faces a water pool that has been naturally generated on a tideland, it does not enter this avoidance area if the swarm is small or sparse. In contrast, if the swarm becomes bigger and forms a dense region, this part of the swarm rushes into the pool without pausing due to the effect of the group. Characteristic (i) suggests perpetual negotiation among individuals with respect to direction. Characteristic (ii) reveals that density affects the mechanism that generates a swarm. Such an inherent noise has been found not only in the swarming of soldier crabs but also in other animal groups. Considering (i) combined with (ii) suggests that inherent noise positively contributes to the generation and maintenance of a swarm. To incorporate soldier crab swarm behaviors into a model, we introduce several potential transitions for each individual that allow the individual to anticipate the movements of other individuals within the swarm.

#### Position update by mutual anticipation

Here we introduce asynchronous position update in our model by mutual anticipation mechanism that is inspired by above characteristics of swarming behavior in soldier crabs, and that is implemented by potential transitions assigned to each individual.

In this model (Fig. 1b), the *i*-th individual at each *t*-th step in a lattice space is assigned its own principal vector *pv*_*i,t*_ (this leads a preferred direction of an individual’s motion) with the lattice length *L*, and has a number of potential transitions that are randomly distributed from the principal vector in a range restricted by the angle *α* (radian) and *L*. We denote the number of the potential transitions by *P*. Here, we briefly describe the mutual anticipation rules (for more detailed description see Supplemental Information, Appendix S1). In the upper left in Fig. 1b, individuals are represented by black lattices. Principal vectors are represented by blue arrows and potential transitions are represented by red arrows with *L* = 2 and *P* = 4.

First, the number of overlapped potential transition targets among individuals is counted as the site popularity regardless of whose potential transition targets these are. If some potential transitions reach a site with popularity greater than one (popular site), an individual moves to the site with the highest popularity. In the upper second left in Fig. 1b, there are two popular sites represented by pink lattices, whose popularities are two. This rule represents the mutual anticipation of the individuals.

For example, people often manage to avoid collisions and walk in a crowd with others using anticipation^40^,^41^,^42^. Therefore, we implement this type of behavior in our model. If more than one individual intends to move to the same site, then one individual whose direction of potential transition reaching the site is the closest to that of its principal vector, is randomly chosen to occupy the site, and the other individuals move to the site with the second highest popularity. The rule of one individual per site is referred to as the repulsion rule due to the asynchronous update. We note that while peoples avoid collisions using anticipation, individuals in our model both intend to move to popular sites and avoid the sites. In other words, if there is no individual that moved to a popular site, individuals whose potential transitions reach the site intend to move there. However, because only one individual is allowed to move to a popular site, after it occupied there, the others avoid moving to the site. In this way, individuals asynchronously both intend to move to and avoid popular sites.

#### Position update by following

Position update by following is implemented by individuals who have not updated their positions because of absence of popular site. In other words, if there is no longer any popular site but is some new empty sites resulting from the flockmate moving to the popular site by mutual anticipation, randomly chosen individuals among those with potential transitions toward the empty sites move to them. Note that the number of individuals who implement following is always limited equal to or smaller than the number of those who implement mutual anticipation. In the right diagram in Fig. 1b, new empty sites are represented by pale blue lattices. This behavior mimics followers and is referred to as the attraction rule.

#### Position update by free moving

Finally, if there is no popular site and no new empty site in the neighborhood, an individual freely moves randomly assuming one of the potential transitions. In the right diagram in Fig. 1b, this is represented by a thick red arrow directed to a white cell. This transition rule results in the final distribution of all individuals shown in the bottom of Fig. 1b.

#### Velocity update

After the positions of all individuals is updated, each individual’s principal vector *pv*_*i*_,_*t*_ is updated as well, based on its own transition in the lattice space *v*_*i,t*_ (i.e., velocity of the *i*-th individual at the *t*-th step) (Fig. 1c). The update is defined as *pv*_*i*_,_*t*+1_ = *L(q*_*i,t*_/|*q*_*i,t*_|), where *u*_*i,t*_ is the unit vector of *v*_*i,t*_ (i.e., *u*_*i,t*_ = *v*_*i,t*_/|*v*_*i,t*_|), *spv*_*i,t*_ is the unit vector of *pv*_*i,t*_ (i.e., *spv*_*i,t*_ = *pv*_*i,t*_/*L*), and *q*_*i,t*_ = *u*_*i,t*_ + *ωspv*_*i,t*_. Note again that the length of the principal vector is *L*. The parameter *ω* is the weight of the previous principal vector (*pv*_*i,t*_), by which the directional persistence of the vector is determined. Therefore, although the direction of the principal vector coincides with that of the velocity when *ω* = 0, it is likely preserved as *ω* increases.

**Figure 2:**
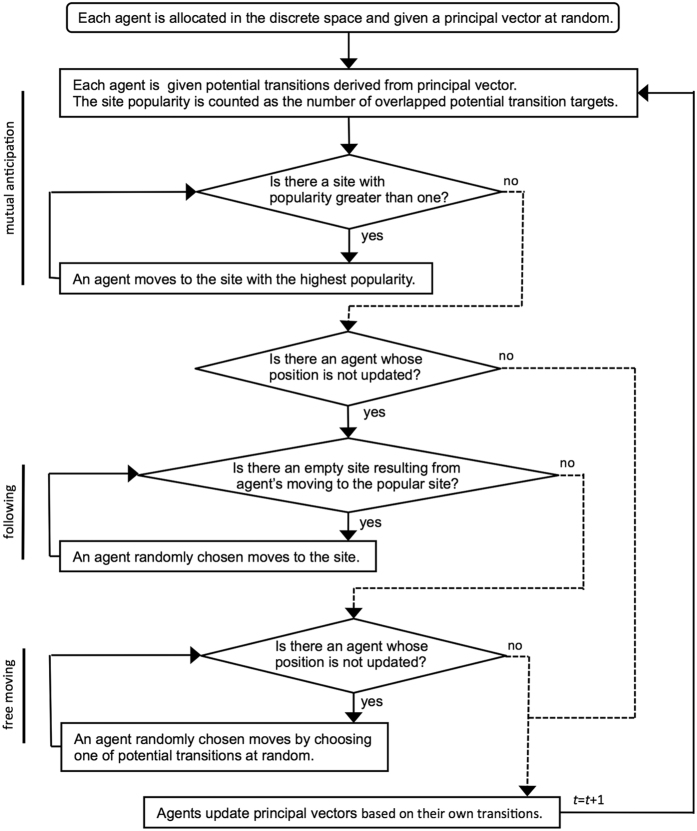
Flow diagram of our model (see also main text and Supplemental Information, Appendix S1).

The main steps of our model are summarized in Fig. 2. Note again that in our model individuals asynchronously move to the next sites, and that because the distribution of popularities of the sites determines the order of update and it could change at each time step, the order of updating could be changed for every time step.

### Polarized swarm derived from mutual anticipation

We demonstrate here the behavior of the swarm based on the mutual anticipation mechanism. Our model based on mutual anticipation contains attraction- and repulsion- like mechanisms but not an alignment rule. Instead, individuals adjust their principal vector based on their previous velocity resulting from mutual anticipation. Consequently, individuals who have principal vectors with randomized direction and who are randomly distributed initially, can compose a coherent swarm with high polarity.

**Figure 3:**
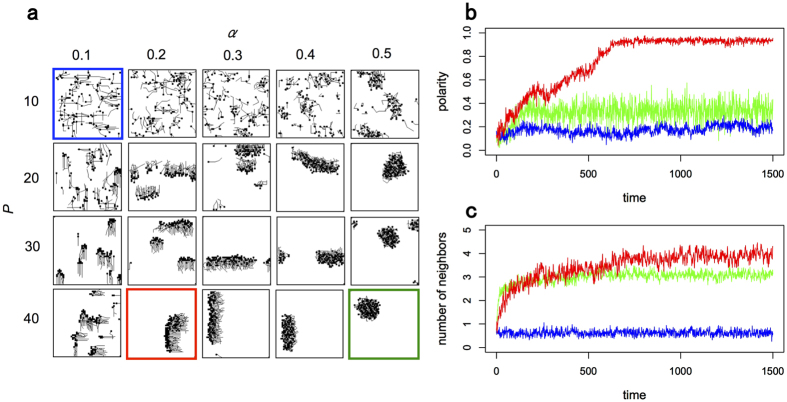
Behavior in the model simulation. (**a**) How changing *P* and *α* affects the patterns of a swarm composed of 100 individuals in a 50 × 50 lattice. Each individual is represented by a square and a 3-step trajectory. (**b**) Polarity plotted against time step. Blue, red and green lines show the polarity obtained by the simulated swarm with *P* = 10 and *α* = 0.1, *P* = 40 and *α* = 0.2, and *P* = 40 and *α* = 0.5, respectively. These colors correspond to those of panel frames in a. (**c**) Density defined as the number of neighbors in the neighborhood, plotted against time step. Correspondence of color lines and simulation setting is the same as (**b**).

Figure 3a demonstrates how various swarming patterns in the model with periodic boundary conditions depend on the parameters *α* and *P* with *L* = 6 and *ω* = 5. In this simulation, the individuals are initially randomly distributed with random principal vectors. Each individual at *t* = 1500 is represented by a black square with a 3-step trajectory tail. If *P* is 10, potential transitions do not give rise to collective motion because ten transitions don’t contribute enough to make a popular site, yielding random transitions. If *P* exceeds 10, mutual anticipation contributes to swarm formation. Particularly, individuals maintain a highly dense and ordered swarm with occasional turbulent motions within the swarm (i.e., movements and changes in the relations between neighbors in an ordered group), if *α* is around 0.2 or 0.3 and *P* is larger than 40. Such inherent turbulent motion in a directed group could occur within bird flock^14^, fish school^15^, and crab swarm^31^. On the other hand, if *α* is too large, because individuals interact with neighbors located almost whole direction, a disordered swarm emerges as if it were a midge swarm^21^.

Figure 3b and c show polarity and density against time in the simulations of Fig. 3a with *P* = 10 and *α* = 0.1 (blue), *P* = 40 and *α* = 0.2 (red), and *P* = 40 and *α* = 0.5 (green), respectively. Polarity is defined by the norm of the average unit velocity between 0 and 1. Density is defined by the average number of individuals in a neighborhood of 3 × 3 lattices. In the case of *P* = 10 and *α* = 0.1, both quantities are remained at a lower value. In the case of *P* = 40 and *α* = 0.2, both quantities increase with time, and they are saturated at a higher value. In the case of *P* = 40 and *α* = 0.5, while density grows with time, polarity is kept at a lower value. Note that we also observed the same results at around *ω* = 5 (for the case of *ω* = 0, 1, …, 10, see Supplementary Information, Figure S3).

### Collective behavior for two individuals

In most of the previous models implementing the alignment rule, each agent interacted with its neighbors by matching the direction of their motions. Hence, if we consider an interaction in a two-individual group, the force to match their directions would increase, when the relative angle between their directions increases. This behavior is in contrast to the observed behavior of real animals in two- or three-individuals groups^13^. Here, we show that the turning response of two-individual behavior in our model, which does not incorporate direct velocity matching, is consistent with the observed turning response in real groups. In the following two-individual simulation, the parameters are *P* = 40, *L* = 6, *ω* = 5, and α = 0.2, as in the above section. In addition, we use a space of 50 × 50 discrete lattices with the periodic boundary conditions. Each simulation consists of 30000 time steps; in the meantime, individuals are fairly close to each other and the polarity is maintained at approximately 0.9.

**Figure 4:**
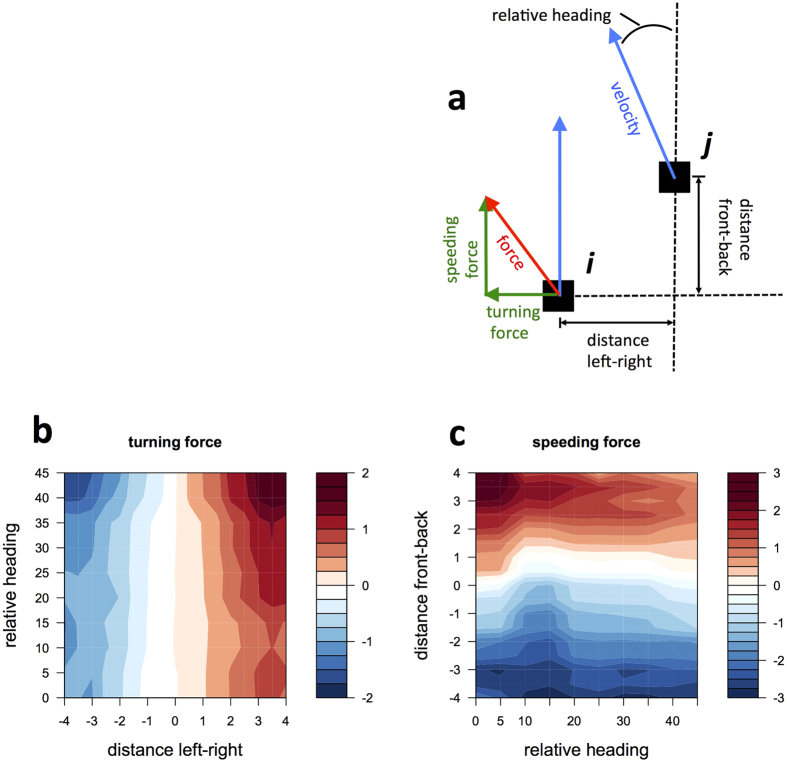
Analysis of the social force within a two-individual group. (**a**) Schematic diagram of dynamical variables. We calculate the relative position and angle of the neighbor. The acceleration as force on the focal individual is decomposed into its speeding and turning components. (**b**) Turning and speeding forces as a function of the relative heading of the two fish and the distance front–back or left–right, respectively.

To analyze the turning response within a two-individual group, we apply a force-based approach as used by Katz *et al*.^13^ (Fig. 4a). In this approach, using *F* = *ma* with *m* = 1, we regard the effective social force^43^ as the acceleration *a* of a focal individual, which is calculated from velocity *v*_*i,t*_. Then, we decompose the force into its speeding force, which is horizontal to the velocity, and its turning force, which is perpendicular to the velocity. In Fig. 4b, we show an individual’s acceleration as a function of its relative heading (relative angle between the focal individual’s and the neighbor’s velocity) and distance left-right (distance between the focal individual’s and the neighbor’s position perpendicular to the focal individual’s velocity). We can see that although turning force becomes stronger as individual are increasingly out of alignment, the direction of turning, however, is always toward the neighbor’s position (i.e., distance left-right) rather than its heading. That is, when the distance left-right increases, the turning force becomes larger, regardless of whether individuals are aligned. In other words, when distance left-right becomes larger, turning force always crosses over to positive value (red in Fig. 4) from negative value (blue in Fig. 4) through zero value (white in Fig. 4). On the other hand, when relative angle becomes larger, turning force can change from lower negative value (pale blue in Fig. 4) to higher one (deep blue in Fig. 4), or from lower positive value (pale red in Fig. 4) to higher one (deep red in Fig. 4), but does not cross over between them. This finding suggests that the turning force depends on the distance left-right rather than the relative angle. This prominent behavior was not predicted by the most previous models with the alignment rule but was observed in real animal groups. This is the first time such behaviors have been simulated and our results are consistent with the experimental results. Moreover, we identify a correspondence between our model and the empirical data with respect to the speeding force that becomes stronger depending on how far the neighbor is in front of or behind the focal individual (distance front-back), rather than the relative heading (Fig. 4c). In other words, when distance front-back becomes larger, speeding force always crosses over to positive value (red in Fig. 4) from negative value (blue in Fig. 4) through zero value (white in Fig. 4). On the other hand, when relative angle becomes larger, speeding force can change from higher negative value (deep blue in Fig. 4) to lower one (pale blue in Fig. 4), or from higher positive value (deep red in Fig. 4) to lower one (pale red in Fig. 4), but does not cross over between them. This is another aspect neglected by many previous models assuming a constant speed.

In addition, when we tested analysis with three individual that was carried out with fish school in ref. 13 on our modeled swarm, we observed that results shown by our model are very similar to experimental data (see Supplemental Information, Figure S4). In short, we found that assuming that individuals average their pairwise interactions as in most previous models describes the main structure of the force well. However, the three-individual swarm actually simulated shows synergistic effects that are stronger than would be expected from averaging the pairwise forces. These results are also consistent with empirical results. As suggested in ref. 13, these synergies that are ignored when interactions are just averaged, functionally may be very important for swarming behavior.

## Discussion

This study presents the first model analysis reproducing the results of a real animal group’s experiment, investigating dynamic individual-level interactions^13^. The experiment sheds light on how the effective social force among individuals depends on their neighbor and/or velocity. To capture the direct behavioral response, the experimenters observed pairwise interactions in the two individuals group. In particular, real animals change their direction depending on their neighbors’ positions rather than the relative angle between their velocities. Nevertheless, many models for collective behavior assumed the alignment rule, by which individuals match their velocity with those of their neighbors. Consequently, these previous models predicted that the direction would increase at a larger relative angle. Furthermore, it is still not clear how this discrepancy between model simulation and experimental results are influenced by the interactions, other than those that follow the explicit alignment rule, between individuals within the group.

Individuals in our model do not follow the explicit alignment rule but act based on mutual anticipation. Each individual in our model has its own principal vector and has potential transitions derived from the principal vector. Through mutual anticipation, the individual moves to a site where potential transitions among individuals are concentrated. Then, the principal vector of individual is adjusted based on the anticipated moves of its neighbors. Hence, although the explicit alignment rule does not apply, interactions with neighbors are reflected in the individual’s decision. As shown in Fig. 3, individuals whose positions and principal vectors are distributed at random in the initial condition can form a coherent swarm with high polarity.

As an emerging dynamic group-level property, our model can reproduce the social dynamics forces of individuals observed in a real animal group^13^. To investigate the social force dynamics, we consider the acceleration of the focal individual as the total force and decompose it into a turning component and a speeding component. If individuals follow the alignment rule, the turning force would increase at a larger relative angle between their velocities. In our model, however, we observe that the strength of the turning force increases depending on how far the neighbor is to the right or left from the focal individual, rather than the relative angle, and is consistent with empirical data. Let us consider simple situations. If a neighbor is far from the focal individual on its sides, then the distance left-right is large, and their potential transitions can overlap only at an intermediate area between them, usually not in front of them. Accordingly, the individuals would move toward the area by mutual anticipation, resulting in a large turning force and a large coherence. In contrast, when the distance left-right is close to zero, the potential overlapping transitions cover a larger area, usually including the area in front of the individuals. Consequently, the individuals can move forward in various ways, resulting in approximately zero average turning force. Moreover, the speeding force increases depending on how far the neighbor is in front of or behind the focal individual rather than the relative angle; this is consistent with the empirical data. Such speeding force dynamics cannot be simulated by models with constant speed. However, models considering mutual anticipation between individuals in different configurations can simulate speeding force dynamics in the same manner as the turning force.

In this study, we demonstrate emergent group-level properties and social force dynamics due to mutual anticipation and not alignment between individuals in a simulated group. This model was inspired by the observation of soldier crab swarms, although similar patterns are clearly present in human crowds^34^. These results suggest that our approach could provide an alternative model for collective behavior.

Finally, we note that there is a possibility that our swarm model defined in a lattice space will lead features not present in the real animal groups. While in our model the direction of individual’s velocity (i.e., its transition in lattice space) cannot change continuously but do discretely, real animals must change their direction continuously.

This difference between real animal group and our lattice model could be important when comparing it with the difference between continuous system (such as Heisenberg spin model) and discrete system (such as Ising spin model) in ferromagnets with respect to a continuous symmetry (in this case, rotational invariance)^44^,^45^,^46^,^47^. Were this an equilibrium problem as in spin system, the explicit symmetry breaking by discrete change of the direction (as in Ising spin system) could lead to an orientationally ordered state which could not occur in the continuous symmetry system (e.g., Heisenberg spin system), as implied by the Mermin-Wagner theorem.

However, it was revealed that ordered state is possible in non-equilibrium moving systems (e.g., swarm systems)^44^. The fundamental difference between swarm systems and spin systems is that individuals in swarm systems, unlike spins, move with respect to another, so that the interaction network is not fixed in time and individuals change their neighbors perpetually^14^,^15^. Hydrodynamic theories of flocking have hypothesized that this moving mechanism reinforces correlations between individuals, enhancing global ordering^45^,^46^,^47^.

Considering the above, while moving swarm systems (of both real animal group and our model) are non-equilibrium, there is a possibility that our discrete model will lead features with respect to ordered state absent in the real animal group. In order to investigate this issue, we will probe continuous space version of our model more sensitively than present discrete version in the future work.

## Methods

### Force map

Here, we define the force map that shows the average turning force of a focal individual as a function of the distance left-right (distance between the focal individual’s and the neighbor’s position vertical to the focal individual’s direction of motion) and the relative angle (see Fig. 4a). To smooth the force map, we used overlapping bins as follows. To compute the mean turning force map <*F*^*i,j*^> as a function of the relative angle and the distance left-right indicated by bin label (*i, j*), the turning component of each acceleration value *a*_*turn*_(*t*) measured at time *t* on the focal individual is first assigned to a bin indexed by (*k(t*), *l(t*)). Here, *k(t*) = [*θ* − mod(*θ*, Δ_*θ*_)]/Δ_*θ*_ and *l(t*) = [*d* − mod(*d*, Δ_*d*_)]/Δ_*d*_, where mod is the modulo operator; *θ* and *d* are the current relative angle and distance left-right, respectively; and bins whose sizes are set at Δ_*θ*_ = 20 and Δ_*d*_ = 2 are spaced by five in the *θ*-axis and by 0.5 in the *d*-axis, so that each data point is assigned to multiple data bins. Then, all data collected in each bin are averaged over all the time steps *t* as follow: <*F*^*i,j*^> = [Σ_*t*_*a*_*turn*_(*t)δ*_*ik(t*)_*δ*_*jl(t*)_]/[Σ_*t*_*δ*_*ik(t*)_*δ*_*jl(t*)_], where *δ*_*ms*_ = 1 if *m* = *n*; otherwise *δ*_*ms*_ = 0. The speeding force map is computed in the same manner.

## Additional Information

**How to cite this article:** Murakami, H. *et al*. Emergence of a coherent and cohesive swarm based on mutual anticipation. *Sci. Rep.* **7**, 46447; doi: 10.1038/srep46447 (2017).

**Publisher's note:** Springer Nature remains neutral with regard to jurisdictional claims in published maps and institutional affiliations.

## Supplementary Material

Supplementary Information
