# Supplementary material for: Emergence of a coherent and cohesive swarm based on mutual anticipation
Source: Sci Rep. 2017 Apr 13;7:46447. doi: 10.1038/srep46447 (PMC5390294; doi:10.1038/srep46447)
Supplement: Supplementary Information [file srep46447-s1.pdf]

# Supplementary Information to “Emergence of a coherent and cohesive swarm based on mutual anticipation”

Hisashi Murakami[1], Takayuki Niizato[2], and Yukio-Pegio Gunji[3]

[1] Department of Information Systems Creation, Kanagawa University, Yokohama, Kanagawa, Japan

[2]Faculty of Engineering, Information and Systems, Tsukuba University, Tsukuba, Ibaraki, Japan

[3]School of Fundamental Science and Engineering, Waseda University, Tokyo, Japan

This SI contains supplementary appendix S1 and figure S1-S4.

## Appendix S1

## Detailed descriptions of our model

Our swarm model consists of $N$ individuals moving in discrete time and in a discrete $S\times S$ space where $S = \{1, 2, ..., s_{\mathrm{MAX}}\}$. The location of the $k$-th individual at the $t$-th step is given by

$$\boldsymbol{P}(k, t) = (x, y) \tag{1}$$

where $x\in S$, $y\in S$, $k\in K=\{1, 2, ..., N\}$, and the boundary condition is periodic. Each $k$-th individual at the $t$-th step has $P$ number of potential vectors $\boldsymbol{v}(k, t; i)$ with $i\in I=\{0, 1, ..., P-1\}$ (Fig. S1). If $i=0$, the vector $\boldsymbol{v}(k, t; 0)$, called the principal vector, is represented by the angle $\theta_{k,t}$, such that

$$\boldsymbol{v}(k, t; 0) = (Int(L\cos\theta_{k,t}), Int(L\sin\theta_{k,t})), \tag{2}$$

where for any real number $x$, $Int(x)$ represents integer $X$ such that $X\leq x<X+1$. $L$ is the length of principal vector. Because of the wrapped fashioned boundary condition, $X\in S$. If $i\neq 0$, the vector is defined using a random value, $\eta$, selected with equal probability from [0.0, 1.0] and a random value (radian), $\xi$, selected with equal probability from $[-\alpha\pi, \alpha\pi]$, as

$$\boldsymbol{v}(k, t; i) = (Int(L\eta \cos(\theta_{k,t}+\xi)), Int(L\eta \sin(\theta_{k,t}+\xi))). \tag{3}$$

The principal vector $\boldsymbol{v}(k, t; 0)$ is a special case where $\eta=1.0$ and $\xi=0.0$. For each $\boldsymbol{v}(k, t; i)$, the target of the vector is represented by $\tau(k, t; i)$ such that

$$\tau(k, t; i) = \boldsymbol{P}(k, t)+ \boldsymbol{v}(k, t; i). \tag{4}$$

To implement mutual anticipation, we define the popularity of the targets of the vectors. The popularity $\zeta(x, y; t)$ is defined for each site at the $t$-th step, $(x, y)$ with $x \in S$, $y \in S$, as the number of the targets of the vectors $\tau(k, t; i)$ such that $\tau(k, t; i) = (x, y)$, that is, the number of the targets pointing to the site. Note that the popularity is defined only for $(x, y)$ such that for any $k\in K$, $\boldsymbol{P}(k, t) \neq (x, y)$, that is, sites that are not occupied by any individuals. This is because the rule of one individual per site is implemented in our model as the repulsion rule due to the asynchronous update (see also main text). Then the definition of the

popularity is summarized by

$$\zeta(x, y; t)$$
$$= |\{\tau(k, t; i), k \in K, i \in I \mid \tau(k, t; i) = (x, y), \text{ and for any } k \in K, \boldsymbol{P}(k, t) \neq (x, y)\}|. \quad (5)$$

Updating the location of individuals is performed asynchronously. If there exists $i \in I$ such that

$$\zeta(\tau(k, t; i)) > 1, \quad (6)$$

the next site for the $k$-th individual is defined by

$$\boldsymbol{P}(k, t+1) = \tau(k, t; s), \quad (7)$$

where $s$ satisfies the condition such that for any $i \in I$,

$$\zeta(\tau(k, t; s)) \geq \zeta(\tau(k, t; i)). \quad (8)$$

In other words, an individual moves to the target of its own potential vector that has maximum popularity. If there multiple sites satisfy condition (8), one site targeted by potential vector whose direction is the closest to that of its principal vector is chosen.

Because updating is asynchronous, a set of sites updated by equation (7) gradually grows. A set of updated sites is represented by $U_N = \{(x, y) \in S \times S \mid \boldsymbol{P}(k, t+1) = (x, y)\}$.

An individual that satisfies condition (6) and moves by equation (7) is called a wanderer. The vacated site generated by a moving wanderer is recorded in memory $\chi(x, y; t)$, which is defined for any $(x, y)$ at the $t$-th step and is initially set to 0 before updating the location, by

$$\chi(x, y; t) = 1, \text{ if } \boldsymbol{P}(k, t) = (x, y) \text{ and } \boldsymbol{P}(k, t+1) \in U_N \quad (9)$$

After all wanderers have been updated, an individual which does not satisfy condition (6) moves to the vacated site to which its own potential vector reaches, by

$$\boldsymbol{P}(k, t+1) = Rd\{(x, y) \in S \times S \mid \chi(x, y; t) = 1, \text{ and for at least one } i \in I, \tau(k, t; i) = (x, y)\},$$

(10)

where $RdJ$ represents an element randomly chosen from set $J$, if $|J|\geq 1$. According to equation (10), a corresponding individual chooses a random site from a set containing sites $(x, y) \in S\times S$ as elements such that they are the vacated sites (i.e., $\chi(x, y; t)=1$) and they are pointed by at least one target of potential vector of the corresponding individual (i.e., for at least one $i \in I$, $\tau(k, t; i) = (x, y)$). An individual whose movement is determined by equation (10) is called a follower.

If an individual is neither wanderer nor a follower, it moves by

$$\boldsymbol{P}(k, t+1) = Rd\{\tau(k, t; i) \mid \text{for any } j\in K', \boldsymbol{P}(j, t)\neq\tau(k, t; i) \text{ and } \tau(k, t; i) \notin U_{\mathrm{N}}\}, \tag{11}$$

where $K'$ is an index set of individuals that are not updated.

Finally, after the position of all individuals is updated, each individual's direction $\theta_{k,t}$ of principal vector $\boldsymbol{v}(k, t; 0)$ is updated as well, based on its own transition in the lattice space (i.e., velocity of the $i$-th individual at the $t$-th step), defined by

$$\boldsymbol{V}(k, t)= \boldsymbol{P}(k, t+1) - \boldsymbol{P}(k, t). \tag{12}$$

The updated direction $\theta_{k,t+1}$ is calculated as the angle of vector $\boldsymbol{q}(k, t)$ defined by

$$\boldsymbol{q}(k, t) = \boldsymbol{V_u}(k, t) + \omega\boldsymbol{v_u}(k, t; 0), \tag{13}$$

where for a vector $\boldsymbol{x}$, $\boldsymbol{x_u}$ represents unit vector of $\boldsymbol{x}$, and $\omega$ is the weight of the previous principal vector by which the directional persistence of the vector is determined (see also main text and figure 1).

The parameters in our model are listed below,

$L$ : the length of principal vector

$P$ : number of potential vectors

$\alpha$ : angle defining the amplitude of angular noise

$\omega$ : the weight of the previous principal vector

## Figure S1

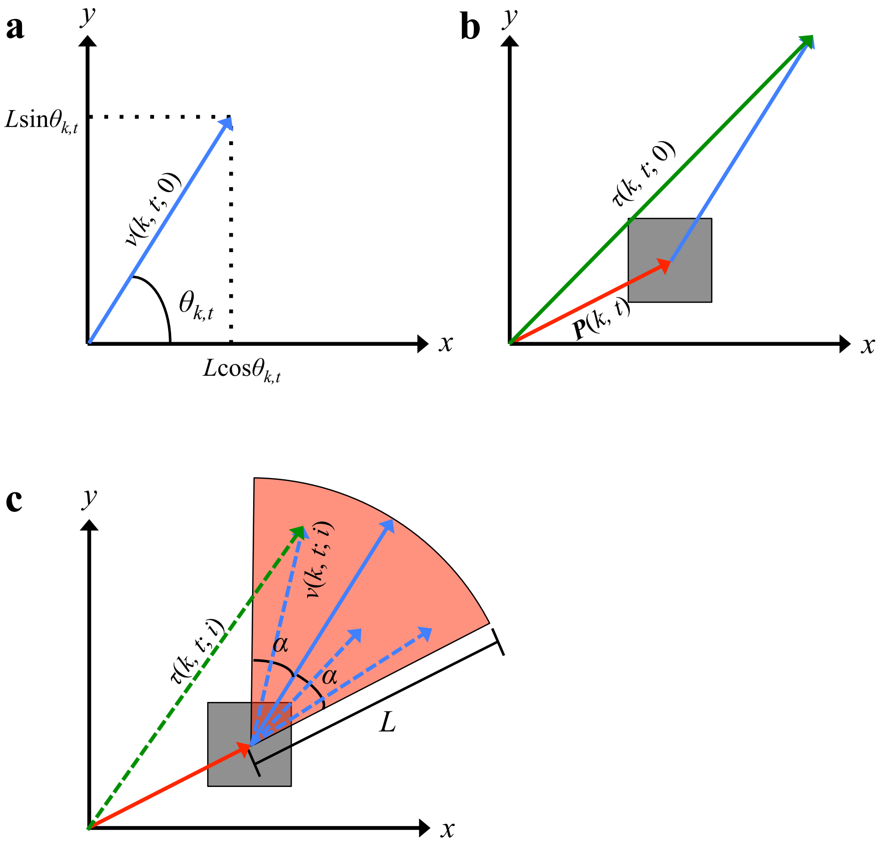


**Figure S1.** Schematic diagrams of principal vector and potential vector in our model. (a) Blue arrow represents the principal vector of $k$-th individual at the $t$-th step $v(k, t; 0)$ defined by equation (2) in appendix S1. (b) Grey square represents the individual. Green arrow represents the target of the principal vector $\tau(k, t; 0)$ defined by a vector composed of the principal vector and the position vector $P(k, t)$ represented by red arrow. (c) Blue dashed arrows represent the potential vectors (one of which is denoted as $v(k, t; i)$) that are randomly distributed from the principal vector in a range restricted by the angle $\alpha$ (radian) and $L$ (pale red area in this figure). Dashed green arrow represents the target of the potential vector $\tau(k, t; i)$. See also main text and appendix S1.

## Figure S2

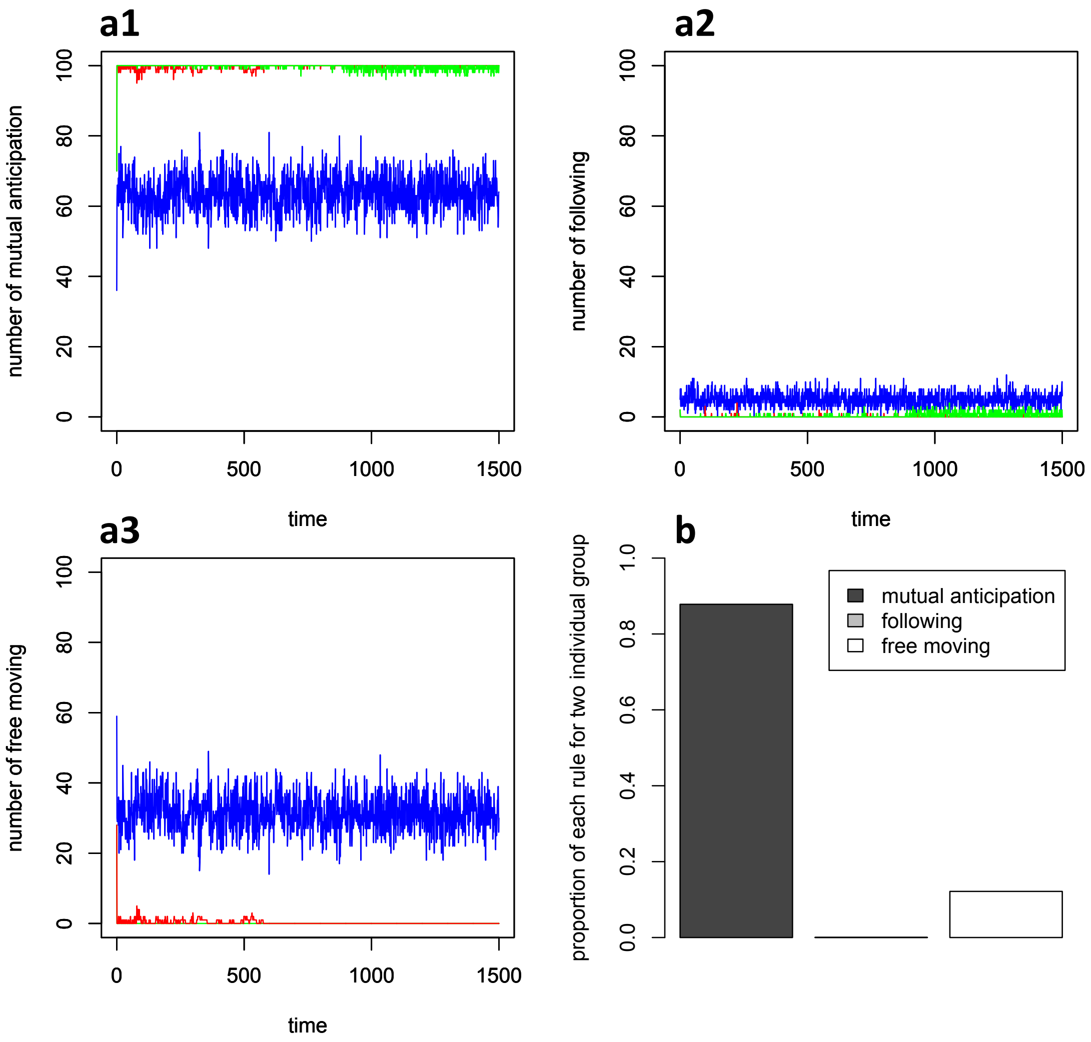


**Figure S2.** Proportion of each rule in our model. (a1) The number of individuals who implement mutual anticipation plotted against time step. (a2) The number of individuals who implement following plotted against time step. (a3) The number of individuals who implement free moving plotted against time step. Correspondence of color lines and simulation setting is the same as Figure 3 in the main text. (b) Proportion of each rule observed in simulation for two-individual group. Simulation setting is the same as Figure 4 in the main text.

## Figure S3

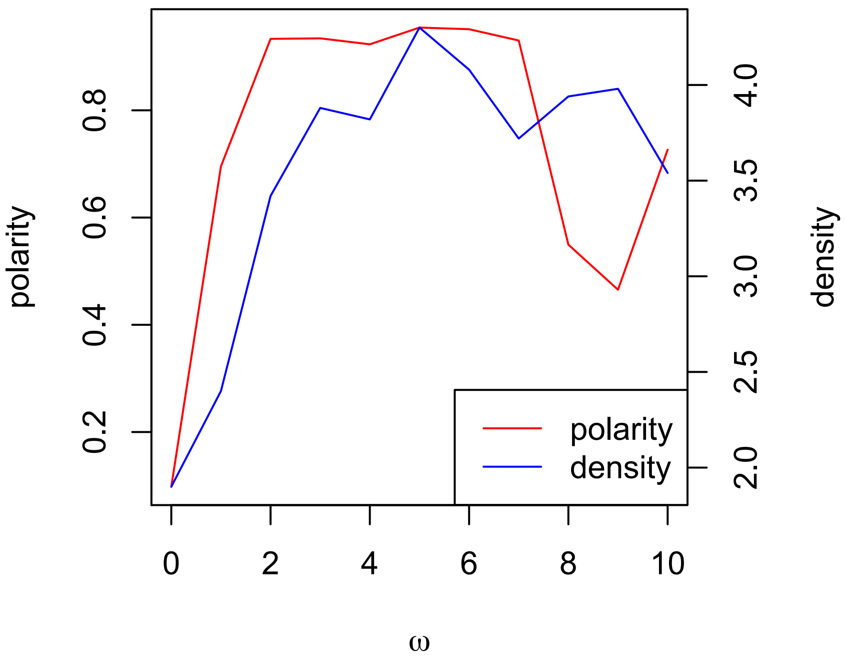


**Figure S2.** Polarity (red) and density (blue) at $t$ = 1500 plotted against ω. The other parameters are set at $P$ = 40, $L$ = 6, and α = 0.2. Polarity is defined by the norm of the average unit velocity between 0 and 1. Density is defined as the number of neighbors in the neighborhood. Around $\omega$ = 5, we observed the same results that was observed at $\omega$ = 5 which was used in the main text, i.e., modeled swarm showed high density with high polarity. On the other hand, if $\omega$ is too small (≤1) or large (≥8), swarm could be not formed densely, showing lower polarity. This is because that if $\omega$ is too small, the direction of the principal vector almost coincides with that of previous principal vector that is initially given at random, and that if $\omega$ is too large, the direction of the principal vector almost coincides with that of the velocity that can be distributed in a widely range restricted by $\alpha$, showing noisy behavior.

# Figure S4

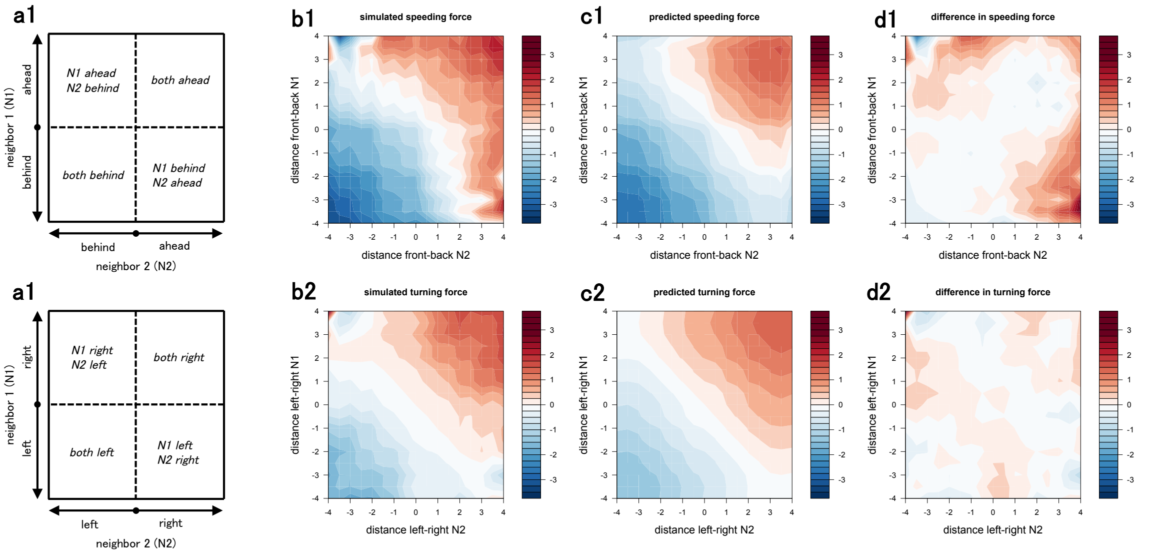


**Figure S3.** Interaction structures in three-individuals groups. (a) Diagram of dynamical variables describing the three-individual configurations. The relative positions of both neighbors are expressed in Cartesian coordinates with the focal individual at the origin. Velocities and effective forces are also expressed in the same way as in the two-individual system. For the speeding forces (Top), the positions of both neighbors are projected onto the axis tangential to the focal individual's direction of motion, and for the turning forces (Bottom), the positions of both neighbors are projected onto the axis perpendicular to the focal individual's direction of motion. (b1 and b2) Measured speeding and turning forces exerted on the focal individual as a function of the distance front–back or left–right to both neighbors, respectively. (c1 and c2) Predicted speeding and turning forces exerted on the focal fish under the hypothesis that fish average pairwise interactions. The maps show results from averaging the two-fish forces presented in Fig. 4. They are symmetric about the diagonal, because the identities of the two neighbors can be interchanged. Note that they display the same qualitative features as those measured for three-individual swarms (b1 and b2), but with significant residual three-body forces, (d1 and d2). (d1 and d2) Residual speeding and turning forces obtained by subtracting panel c1 from b1 and c2 from b2. Assuming that individuals average their pairwise interactions successfully captures the basic structure of the force. However, the residual forces show a substantial three-body effect producing stronger effective forces when the focal individual is between both neighbors.
